# Urinary Eosinophil Protein X in Children with Atopic Asthma

**DOI:** 10.1155/2007/49240

**Published:** 2007-06-13

**Authors:** M. Nuijsink, W. C. J. Hop, P. J. Sterk, E. J. Duiverman, P. S. Hiemstra, J. C. de Jongste

**Affiliations:** ^1^Department of Paediatric Respiratory Medicine, Juliana Children's Hospital, P.O. Box 60605, 2506 LP The Hague, The Netherlands; ^2^Department of Epidemiology and Biostatistics, Erasmus University Medical Centre, P.O. Box 2040, 3000 CA Rotterdam, The Netherlands; ^3^Department of Pulmonology, Academic Medical Center University of Amsterdam, P.O. Box 22660, 1100 DD Amsterdam, The Netherlands; ^4^Department of Paediatric Respiratory Diseases, Beatrix Children's Hospital, The University Medical Centre Groningen, P.O. Box 30001, 9700 RB Groningen, The Netherlands; ^5^Department of Pulmonology, Leiden University Medical Centre, P.O. Box 9600, 2333 ZA Leiden, The Netherlands; ^6^Department of Paediatric Respiratory Medicine, Sophia Children's Hospital, Erasmus University Medical Centre, P.O. Box 2060, 3000 CB Rotterdam, The Netherlands

## Abstract

The aim of this study was to investigate the relationship between urinary eosinophil protein X (uEPX) and asthma symptoms, lung function, and other markers of eosinophilic airway inflammation in asthmatic school children. *Methods*. A cross-sectional study was performed in 180 steroid dependent atopic children with stable moderately severe asthma, who were stable on 200 or 500*μ*g of fluticasone per day. uEPX was measured in a single sample of urine and was normalized for creatinine concentration (uEPX/c). Symptom scores were kept on a diary card. FEV_1_ and PD_20_ methacholine were measured. Sputum induction was performed in 49 and FE_NO_ levels measured in 24 children. *Results*. We found an inverse correlation between uEPX/c and FEV_1_ (*r* = −.20, *P* = .01) and a borderline significant correlation between uEPX/c and PD_20_ methacholine (*r* = −.15, *P* = .06). Symptom score, %eosinophils and ECP in induced sputum and FE_NO_ levels did not correlate with uEPX/c. *Conclusion*. uEPX/c levels did not correlate with established markers of asthma severity and eosinophilic airway inflammation in atopic asthmatic children.

## 1. INTRODUCTION

Eosinophilic airway inflammation is the pathological substrate of allergic asthma both in adults and in children [1, 2]. The severity of airway inflammation correlates poorly with symptoms and lung function [3]. As asthma treatment with inhaled steroids aims at reducing inflammation, there is a need to monitor the disease with a marker of inflammation [4, 5]. Potential markers are serum eosinophilic cationic protein (ECP), induced sputum cellularity and soluble markers [6], and the concentration of nitric oxide in exhaled air (FE_NO_) [7, 8].

Eosinophil protein X (EPX) is one of the toxic proteins present in eosinophil granules and is released by activated eosinophils. EPX can be measured accurately in urine (uEPX) [9]. Therefore, uEPX can be regarded as a marker of eosinophil degranulation in vivo [10]. uEPX levels in allergic asthmatic children were found to be significantly higher than in healthy controls [11–14]. Treatment with inhaled steroids reduced uEPX levels [14]. We hypothesized that measuring EPX in urine could potentially prove to be useful for monitoring eosinophilic airway inflammation in children and may complement other markers of asthma control such as symptom scores and lung function.

The aim of this study was to evaluate the relationship between uEPX and current symptoms and lung function parameters, and the relation between uEPX, induced sputum eosinophilia, and FE_NO_. For this purpose, we analyzed cross-sectional data obtained at enrolment for a multicentre trial.

## 2. METHODS

### 2.1. Subjects

Data were obtained from steroid-dependent asthmatic children who took part in a large randomized controlled multicentre trial (CATO: Children Asthma Therapy Optimal). One hundred and eighty atopic (RAST ≥ class 1 for at least one airborne allergen) children, median age 10.3 years (range 6–16 years), with a documented clinical history of moderately severe asthma were recruited from paediatric clinics in 8 general hospitals and 7 university hospitals in The Netherlands. All had been treated with inhaled corticosteroids (ICS) for at least 4 weeks. Data were obtained during a clinic visit at the end of the run in period of 4–12 weeks. During this period, they were treated with fluticasone dipropionate 200 *μ* *g*/*d* (*n* = 102) or 500 *μ* *g*/*d* (*n* = 78). All parents and children if >12 years gave their written informed consent. The study was approved by the medical ethics committees of all participating hospitals.

### 2.2. Symptom scores

Two weeks before visiting the hospital, patients kept a diary in which symptoms (shortness of breath, wheeze, and cough) were scored twice a day each on a 4-point (0–3) scale. Cumulative symptom scores were calculated over 14 days (maximum score 252).

### 2.3. Fractional exhaled nitric oxide

The fractional concentration of exhaled nitric oxide (FE_NO_) was measured with the online single breath method, using the NIOX NO-analyzer (Aerocrine, Stockholm, Sweden) according to ERS/ATS guidelines [15].

As FE_NO_ could only be measured in 1 participating university centre, only part of the children underwent FE_NO_ measurements.

### 2.4. Flow-volume curves

Flow-volume curves and forced expiratory volume in 1 second (FEV_1_) were measured on a dry rolling seal spirometer according to recommendations [16]. Results are expressed as percentage of predicted values [17].

### 2.5. Bronchial challenge test

Bronchial responsiveness was determined by a methacholine challenge [18]. PD_20_ methacholine (provocative dose of methacholine causing FEV_1_ fall 20% from baseline) was assessed by linear interpolation of the last two points of the log dose-response curve where FEV_1_ had fallen below 20% of baseline value.

### 2.6. Sputum induction and processing

Sputum induction was performed by 5 university centres and 3 paediatric clinics in general hospitals. Sputum was induced according to a standardized method by inhaling an aerosol prepared from hypertonic sodium chloride 4.5% w/v [19, 20]. Differential cell counts of the cytospins were performed by counting 500 cells. Sputum samples containing more than 80% squamous cells were excluded from the analysis [20].

In sputum supernatant, ECP was measured by fluoroenzyme immunoassay (Pharmacia, Uppsala, Sweden).

### 2.7. Urinary eosinophil protein X

A spot sample urine was collected from each individual at the clinic visit and immediately stored at −20°C. uEPX was determined using a commercial enzyme-linked immunosorbent assay (ELISA) for human EPX in 50-fold diluted samples according to the manufacturers recommendations (Medical and Biological Laboratories, Naka-Ku Nagoya, Japan). The sensitivity of the assay was 0.62ng/mL. Urinary creatinine levels were measured by using the alkaline picrate method (Jaffé reaction) (Roche, Mannheim, Germany). Urinary EPX concentrations were expressed as *μ* *g* per mmol creatinine (uEPX/c).

### 2.8. Data analysis

All variables with a non-Gaussian distribution (symptom score, PD_20_ methacholine, FE_NO_, % eosinophils in sputum, ECP in sputum, and uEPX) could be normalized by log-transformation. The significance of the relation between uEPX and lung function variables or other markers of inflammation was calculated using Spearman's rank correlation coefficients. A two-sided *P* value of <.05 was considered statistically significant.

## 3. RESULTS

One hundred and eighty subjects (105 boys (58.3%)) participated. Asthma was controlled by fluticasone dipropionate 200 *μ* *g*/day (*n* = 102) or 500 *μ* *g*/day (*n* = 78).

All subjects performed spirometry and recorded symptoms in a diary. Six children inhaled short-acting *β*-agonists prior to the visit, their results were excluded from analysis. One hundred and seventy eight children performed a bronchial challenge test; two had FEV_1_ < 80% of personal best and were therefore not tested. Children who had used *β*-agonist within 8 hours before the test (*n* = 6) were again excluded. For logistic reasons, sputum induction was done in part of the subjects. Forty nine of the 98 sputum inductions yielded adequate sputum samples (50%). At randomization, only one university centre had the facility to measure FE_NO_ (*n* = 24 subjects).

Baseline results of lung function, symptom score, and markers of inflammation are given in Table 1. uEPX/c showed a log-normal distribution, median 185 *μ* *g*/mmol creatinine (range 2–3114 *μ* *g*/mmol creatinine). UEPX/c did not correlate with age and was not different between boys and girls.

### 3.1. Relation between uEPX/c and clinical markers of asthma severity (Table 2)

**Figure 1:**
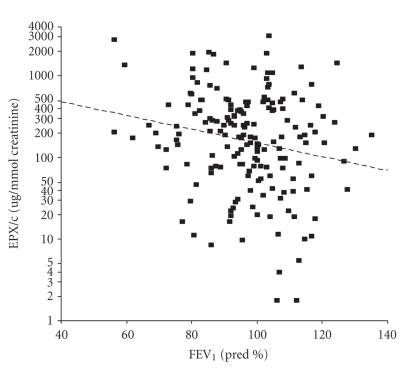
Scatter plot of urinary eosinophil protein X per mmol creatinine (uEPX-c) versus forced expiratory volume in 1 second (FEV_1_), *n* = 174.

UEPX/c did not correlate with symptom scores or inhaled steroid dose. There was a significant inverse correlation of uEPX/c with FEV_1_(*r* = −.18, *P* = .02) (Figure 1). The association between uEPX/c and FEV_1_ did not significantly differ between children using 200 *μ* *g* fluticasone per day and those using 500 *μ* *g* (Anova, *P* = .19). For each 10% points increase of FEV_1_ (pred. %) the geometric mean EPX/c ratio decreases 18% (95% CI: 5,30%). The correlation between uEPX/c and PD_20_ methacholine was borderline significant (*r* = −.14, *P* = .08).

### 3.2. Relation between uEPX and markers of asthmatic airway inflammation (Table 2)

uEPX/c did not correlate with the % eosinophils or ECP in induced sputum, or with FE_NO_. Relations between uEPX and PD_20_ methacholine or markers of asthmatic airway inflammation did not significantly differ when analysis was adjusted for fluticasone dose.

Correlations were similar when children with eczema were excluded from the analysis.

## 4. DISCUSSION

We found a significant correlation of uEPX/c and FEV_1_, and no association between uEPX/c and bronchial responsiveness or symptom scores in a large group of children with moderately severe allergic asthma. In subgroups, no significant correlations between uEPX/c and other markers of eosinophilic airways inflammation (% eosinophils and ECP in induced sputum or FE_NO_) were found.

This is the first study reporting uEPX/c levels in relation with markers of asthma severity and inflammation in a large population of children with atopic asthma, treated with inhaled steroids. Lugosi et al. have shown that uEPX levels were increased in symptomatic versus nonsymptomatic children with asthma, treated with inhaled steroids or disodium cromoglycate [21], and Oosaki et al. [22] found significantly elevated uEPX levels during acute asthma exacerbationss in children. All subjects included in our study had stable well-controlled asthma, as evidenced by a median cumulative symptom score of only 17 of a maximum of 252. Conflicting data have been published on the association between uEPX/c and pulmonary function tests [21, 23]. We found a significant negative correlation between FEV_1_ and uEPX/c. It should be mentioned that the scatter was wide, and individual uEPX/c therefore varied widely for a given FEV_1_ level. Hence, such correlations are unlikely to be detected in smaller groups. However, the within-subject variation of both parameters in time had not been studied. We confirmed our hypothesis that uEPX/c and bronchial hyperresponsiveness are not closely correlated. Lack of correlation between the severity of bronchial hyperresponsiveness and uEPX/c levels was also reported in 3 previous studies [24–26]. A close correlation between bronchial hyperresponsiveness and uEPX/c was not expected, because bronchial hyperresponsiveness is multifactorial and is not only caused by (eosinophilic) airways inflammation, but also by airway geometry, airway remodelling, and autonomic dysregulation.

Our hypothesis that uEPX/c would correlate with markers of eosinophilic airway inflammation could not be confirmed, as we found no correlation between uEPX/c and the percentage eosinophils in induced sputum. Others likewise found no correlation between uEPX/c and bronchoalveolar lavage cell counts in adult asthmatic patients [10]. An alternative explanation for not finding significant correlations between percentages of sputum eosinophils and uEPX/c could be that uEPX is only released by activated eosinophils, whereas in sputum we counted activated as well as nonactivated eosinophils. Also, the number of children from whom suitable sputum samples or FE_NO_ values were obtained was relatively small.

We found no correlation between uEPX/c and sputum ECP levels. In contrast, Mattes et al. [11] reported a positive correlation between uEPX/c and sputum ECP in 25 stable asthmatic children on inhaled corticosteroids. They found much higher ECP concentrations than we did (median 453ng/mL, range 40–2600; and 29ng/ml, 1-2345, resp.). The reason for this is not clear, but may be related to different sputum processing techniques.

One could argue that the lack of correlation between uEPX/c and percentage of sputum eosinophils, or ECP levels in sputum supernatant, could be due to the wide scatter of uEPX. However, all urine samples were immediately stored at −20°C and uEPX and urinary creatinine levels measurements were performed in a central laboratory (Leiden University Medical Hospital) to reduce variability in the analysis. All EPX measurements were done in duplicate and the within-subject reproducibility of uEPX levels was good.

It has been reported that in atopic dermatitis, concentrations of eosinophil- specific mediators, including uEPX/c, are increased [27, 28]. However, we found that the presence or absence of atopic eczema did not influence the correlations between uEPX/c and the percentage eosinophils or ECP in induced sputum. We cannot exclude that heterogeneity of study groups with respect to other atopic disorders than asthma could have affected the correlation between uEPX/c and other markers of eosinophilic airways inflammation.

At the onset of our study, a circadian rhythm of uEPX/c had not been reported. Urine samples were not all obtained at the same time of the day. Since the start of our study, it became evident that a circadian rhythm of uEPX/c with lowest levels at 7 p.m. and highest at 7 a.m. in both asthmatic and healthy controls exists [23, 29–31]. Hence, diurnal variability may have introduced scatter of uEPX, thus weakening a possible correlation.

Two previous studies reported significant positive correlations between uEPX/c and FE_NO_ in corticosteroid-dependent childhood asthma [11, 29]. We found no significant correlation between uEPX/c and FE_NO_ in a small subgroup of the study population. For FE_NO_, no important circadian variation was found, employing the same measurement technique that we have used [32], but conflicting results have also been published [27, 33]. A possible circadian rhythm might have affected FE_NO_ and weakened any cross-sectional relationship.

In conclusion, the present data show a weak inverse correlation between uEPX/c and FEV_1_, and a borderline correlation between uEPX/c and PD_20_ methacholine. No significant correlation was found between uEPX/c and markers of eosinophilic airway inflammation including % eosinophils or ECP levels in induced sputum or FE_NO_. The number of children performing FE_NO_ was small, therefore this correlation should be interpreted with caution. Our findings are not encouraging for uEPX/c as a complementary marker of airway inflammation in asthma. As to whether uEPX/c can be useful as a marker for monitoring asthma management in children is worth prospectively looking at.

## Figures and Tables

**Table 1:** Characteristics of study subjects. Values are median (range). FEV_1_ is forced expiratory volume in 1 second; PD_20_ methacholine is provocative dose of methacholine causing FEV_1_ fall 20% from baseline; ECP is eosinophil cationic protein; FE_NO_ is fractional concentration of nitric oxide in exhaled air; uEPX/c is urinary eosinophil protein X per mmol creatinine.

	Fluticasone dose		Total
	200 *μ* *g*/day	500 *μ* *g*/day	–

Age (years)	10(96.4 − 16.8)	11.3(6.4 − 16.7)	10.3(6.4 − 16.8)
	*n* = 102	*n* = 78	*n* = 180

Gender (m/f)	60/40	45/33	105/75

FEV_1_ (pred. %)	99(56 − 135)	96(56 − 96)	97(56 − 135)
	*n* = 101	*n* = 73	*n* = 174

Cumulative symptom score	18.5(0 − 113)	14(0 − 152)	17.0(0 − 152)
	*n* = 102	*n* = 78	*n* = 180

PD_20_ methacholine (*μ*g)	200(3− > 1570)	48(1− > 1570)	68(1− > 1570)
	*n* = 100	*n* = 72	*n* = 172

Eosinophils sputum (%)	1(0 − 72)	1(0 − 43)	1.0(0 − 72)
	*n* = 29	*n* = 20	*n* = 49

ECP sputum (ng/ml)	17(0 − 2345)	38(0 − 538)	29(0 − 2345)
	*n* = 24	*n* = 19	*n* = 43

FE_NO_ (ppb)	11(5 − 63)	9(1 − 29)	10(1 − 63)
	*n* = 12	*n* = 12	*n* = 24

uEPX/c (*μ*g/mmol)	189(2 − 2828)	180(10 − 3114)	185(2 − 3114)
	*n* = 102	*n* = 78	*n* = 180

**Table 2:** Correlations between uEPX or uEPX-c and clinical markers of asthma severity or markers of asthmatic inflammation. *r* values were all analyzed by Spearman's rank correlation tests. uEPX/c is urinary eosinophil protein X per mmol creatinine; FEV_1_ is forced expiratory volume in 1 second; PD_20_ methacholine is provocative dose of methacholine causing FEV_1_ fall 20% from baseline; ECP is eosinophil cationic protein; FE_NO_ is fractional concentration of nitric oxide in exhaled air.

Variable	*N*	Log uEPX/c	
		*r*	*P*

Age	180	−.01	.90
Symptom score	180	.03	.72
FEV_1_	174	−.18	.02
PD_20_ methacholine	172	−.14	.08
% eosinophils in sputum	49	.17	.26
ECP sputum	43	−.03	.83
FE_NO_	24	.16	.46

## APPENDIX

This work has been prepared by the authors on behalf of the CATO Study Group.

*CATO Study Group Members*

M. Nuijsink M.D. and J. M. Kouwenberg M.D. (Department of Paediatric Respiratory Medicine, Juliana Children's Hospital, The Hague). W. C. J. Hop Ph.D. (Department of Epidemiology and Biostatistics, Erasmus University Medical Centre, Rotterdam). Professor P. J. Sterk Ph.D. and E. R. V. M. Rikkers-Mutsaerts M.D. (Academic Medical Center, Amsterdam). Professor E. J. Duiverman M.D. Ph.D. (Department of Paediatric Respiratory Diseases, University Medical Centre Groningen, University Groningen). Professor J. C. de Jongste M.D. Ph.D. (Department of Paediatric Respiratory Medicine, Erasmus University Medical Centre/Sophia Children's Hospital, Rotterdam). O. H. van der Baan-Slootweg M.D. and E. E. M. van Essen-Zandvliet M.D. Ph.D. (Asthma Center Heideheuvel, Hilversum). N. J. van den Berg M.D. (Flevo Hospital, Almere). Professor Dr. J. M. Boogaard Ph.D. P. L. P. Brand M.D. Ph.D., R. J. Roorda M.D. Ph.D., and A. W. A. Kamps M.D. Ph.D. (Isala Klinieken, Zwolle). G. Brinkhorst M.D. (Medical Center, Alkmaar). J. E. Dankert-Roelse M.D. Ph.D. and A. F. Nagelkerke M.D. (VU Medical Center, Amsterdam). R. van Gent M.D. (Maxima Medical Center, Veldhoven). J. W. C. M. Heijnens M.D. (Maasland Hospital, Sittard). J. J. E. Hendriks M.D. Ph.D. and Q. Jöbsis M.D. Ph.D. (University Hospital Maastricht). J. van der Laag M.D. and H. J. L. Brackel M.D. Ph.D. (University Medical Center/Wilhelmina Children's Hospital, Utrecht). J. C. van Nierop M.D. (Academic Medical Centre, Amsterdam). A. A. P. H. Vaessen-Verberne M.D. Ph.D. (Amphia Hospital, Breda).
